# Novel Chlorin with a HYNIC: Synthesis, ^99m^Tc-Radiolabeling, and Initial Preclinical Evaluation

**DOI:** 10.3390/molecules30010117

**Published:** 2024-12-31

**Authors:** Alexander Popov, Nikita Suvorov, Mariia Larkina, Evgenii Plotnikov, Ruslan Varvashenya, Vitalina Bodenko, Gleb Yanovich, Petr Ostroverkhov, Maxim Usachev, Elena Filonenko, Mikhail Belousov, Mikhail Grin

**Affiliations:** 1 M. V. Lomonosov Institute of Fine Chemical Technology, MIREA—Russian Technological University, 86 Vernadsky Av., 119571 Moscow, Russia; suvorov.nv@gmail.com (N.S.); mrp_ost@mail.ru (P.O.); maximus021989@mail.ru (M.U.); elena.filonenko@list.ru (E.F.); michael_grin@mail.ru (M.G.); 2 Science and Education Laboratory for Chemical and Pharmaceutical Research, Siberian State Medical University, 634050 Tomsk, Russia; marialarkina@mail.ru (M.L.); plotnikovev@tpu.ru (E.P.); mr.varvashenya@mail.ru (R.V.); bodenkovitalina@gmail.com (V.B.); sonne_gleb@mail.ru (G.Y.); 3 Research Centrum for Oncotheranostics, Research School of Chemistry and Applied Biomedical Sciences, Tomsk Polytechnic University, 634050 Tomsk, Russia; 4 P. A. Hertsen Moscow Oncology Research Center, 125284 Moscow, Russia; 5 Department of Pharmaceutical Analysis, Siberian State Medical University, 634050 Tomsk, Russia; mvb63@mail.ru

**Keywords:** technetium-99m, chlorin, radiopharmaceuticals, HYNIC, cancer

## Abstract

The use of radiopharmaceuticals for diagnostics in oncology allows for the detection of the disease at an early stage. Among diagnostic radionuclides, ^99m^Tc is a promising isotope that has been used to create several drugs for clinical use. One of the most effective ^99m^Tc chelators is 6-hydrazinylnicotinic acid (HYNIC), which, when combined with various vector molecules, can be used for targeted delivery of radionuclides to tumor tissues. At the same time, it is known that tetrapyrrole macrocycles are capable of selective accumulation in tumors, and thus can be used to target radiopharmaceuticals with ^99m^Tc. In this work, the conjugate of natural chlorin and HYNIC was obtained, and preliminary preclinical studies were carried out on its radiocomplex with ^99m^Tc.

## 1. Introduction

The use of radiopharmaceuticals for the treatment and diagnosis of oncological diseases allows for establishment of a diagnosis at an early stage and for carrying out adequate treatment more effectively [1]. The vast majority of currently available diagnostic radiopharmaceuticals is used in positron emission tomography (PET) or single-photon emission computed tomography (SPECT) [2]. Among the diagnostic radionuclides used for radiolabeling, the use of a ^99m^Tc-radioactive label is an attractive option. The ^99m^Tc radionuclide has a favorable nuclear decay characteristic providing high spatial resolution and low absorbed dose to patients, cost-effective accessibility through ^99^Mo/^99m^Tc radionuclide generators, and widespread clinical implementation [3,4].

A large number of ligands with different structures have been proposed for the chelation of ^99m^Tc [5,6,7,8], which can be used individually [9,10] or as part of conjugates with various biologically active molecules [11]. One of the most efficient bifunctional chelating agents for ^99m^Tc is 6-hydrazinylnicotinic acid (HYNIC). It can form complexes with ^99m^Tc via the nitrogen atoms in pyridine and hydrazine moiety. Since HYNIC is unable to saturate the technetium-99m coordination sphere alone, the coordination sphere should be supplemented by adjunct co-ligands, such as ethylenediaminediacetic acid (EDDA) and tricine, as an intermediate co-ligand enables the formation of a stable complex with a high radiochemical yield [12,13]. Routine clinical use may benefit from this approach’s simplicity and low time consumption. Various targeted molecules containing HYNIC have been described in the literature, which are high-molecular compounds or small targeted molecules, and some of them are already being used in clinical practice [14,15,16].

The ability of tetrapyrrole macrocycles to selectively accumulate in tumors compared to healthy tissues is well known [17,18,19].

These compounds bind to and accumulate in tumor cells through various mechanisms, such as the “enhanced permeability and retention (EPR) effect”, binding to low-density lipoproteins [20], capture by macrophages, and increased solubility at low pH [21].

This feature of biodistribution forms the basis for the use of these compounds in photodynamic therapy and fluorescence diagnostics of cancer as photosensitizers [22,23,24]. A special place among these compounds is occupied by derivatives of natural chlorophyll A, which exhibit high selectivity of accumulation in tumor tissues and lack toxic effects on the body due to their rapid biodegradability [25,26]. The source of these photosensitizers is algae called *Spirulina platensis*, which contains chlorophyll A. This raw material is relatively inexpensive, and the methods for isolating and synthesizing natural chlorin have been well researched [27,28].

Certain porphyrins, chlorins, and their derivatives can form metal complexes that are both thermodynamically and kinetically stable. These compounds also show the ability to specifically target tumors, regardless of whether a coordinating metal ion is present in their central nucleus. However, few studies have been published on radioactively labeled photosensitizers that utilize tetrapyrrole macrocycles [13,29,30,31,32].

Predominantly labeled porphyrins have been studied due to their unique structural features and geometry of the tetrapyrrole fragment. This structure enables the chelation of certain small-radius metals, including ^99m^Tc, ^188^Re, ^68^Ga, and ^111^In. However, radioactively labeled ^111^In porphyrin structures based on tri(sulfonyl-phenyl) and tri(methyl-pyridinium) porphyrins have shown a significant tendency to localize in non-target organs [29].

The complex of ^188^Re labeled 5,10,15,20-tetra(4-pyridyl)-21H,23H-porphyrin demonstrated a strong affinity for Ehrlich ascites carcinoma and exhibited a high tumor-to-blood ratio [30]. The hydrophilic complexes 5,10,15,20-tetrakis(pentafluorophenyl)porphyrin (^68^Ga-TFPP) and [^67^Ga]-TFPP demonstrated high tumor uptake in fibrosarcoma, along with favorable tumor-to-blood and tumor-to-muscle ratios in preclinical studies. Additionally, the complex 5,10,15,20-tetrakis(2,4,6-trimethoxyphenyl)porphyrin (^68^Ga-TTMPP) exhibited specific localization in the fibrosarcoma tumor region and also showed high tumor-to-muscle ratios. Its polar nature contributes to rapid distribution and elimination from the body [31].

The aim of this work was the synthesis of natural chlorin that contains a fragment of 6-hydrazinylnicotinic acid. The incorporation of the HYNIC ligand into the chlorin molecule structure enables one-step labeling using TcO_4_^−^ technetium pertechnetate (from a ^99^Mo/^99m^Tc generator eluate). Its ^99m^Tc-radiolabeling and primary preclinical studies were conducted to evaluate the potential for use in nuclear medicine imaging. The study evaluated the dose-dependent effects of the ^99m^Tc-labeling HYNIC-chlorin complex on biodistribution in Nu/J mice with epidermoid carcinoma xenografts. Doses of 6 mg/kg and 12 mg/kg were deemed close to the expected therapeutic dose, while a dose of 1.2 mg/kg was considered below the required therapeutic level.

## 2. Results

### 2.1. Synthesis of HYNIC-Chl

**Scheme 1:**
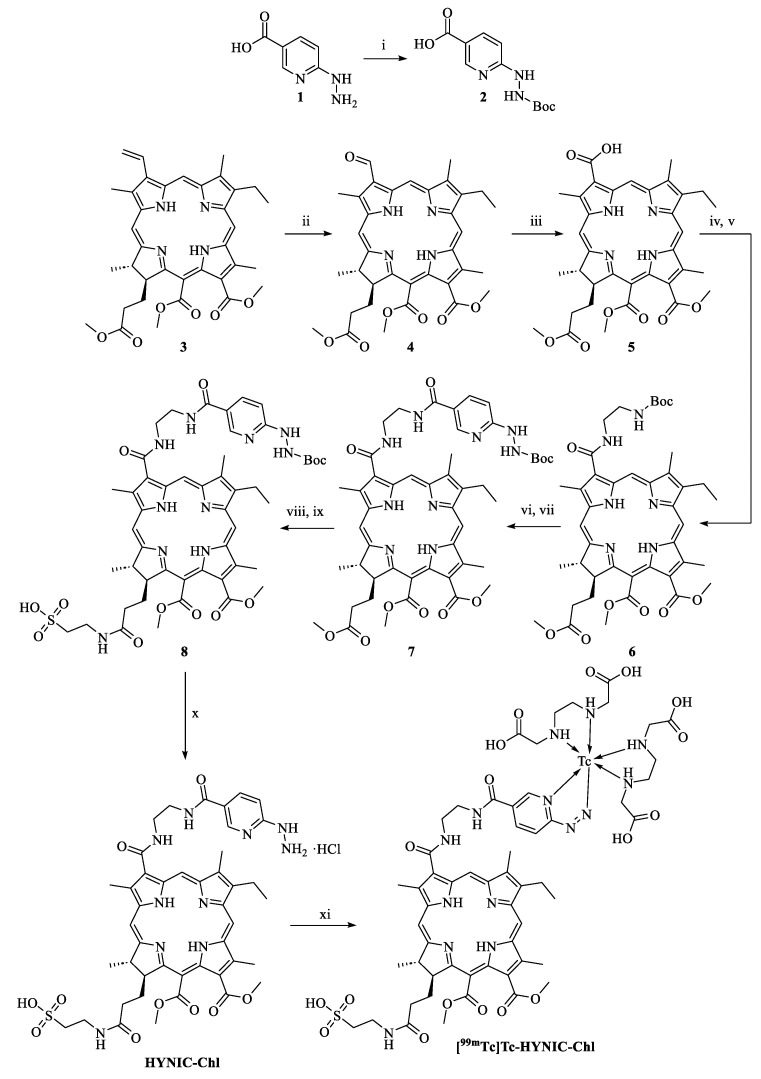
Reagents and conditions: i—Boc_2_O, Et_3_N, DMF, 25 °C, 17 h; ii—OsO_4_/CH_2_Cl_2_, NaIO_4_, THF/H_2_O, 25 °C, 2 h; iii—NaClO_2_, NH_2_SO_3_H, DMSO, THF/H_2_O, 25 °C, 2 h; iv—HBTU, DIPEA, DMF, 25 °C, 30 min; v—N-Boc-ethylenediamine, DMF, 25 °C, 5 min; vi—CF_3_COOH/CH_2_Cl_2_, 25 °C, 10 min; vii—2, HBTU, DIPEA, DMF, 25 °C, 20 min; viii—KOH, H_2_O, THF, 25 °C, 6 h; ix—taurine, HBTU, DIPEA, DMF, 25 °C, 1 h; and x—0.1 M HCl, 120 °C, 2 h; xi—DMSO, water, tricine, EDDA, SnCl_2_, HCl, [^99m^Tc]TcO_4_^−^, 85 °C, 15 min.

The structure of chlorin p_6_ trimethyl ester allows for chemical modifications of the pyrrole A of the chlorin macrocycle, as well as the aliphatic ester group at position 17. In the initial phase of the synthesis process (Scheme 1), the vinyl group of pyrrole A underwent oxidation, resulting in the formation of a carboxylic group. We attempted to implement this change by employing a variety of established techniques for the oxidation of alkenes (Oxone/OsO_4_ [33], NaIO_4_/RuCl_3_ [34], Oxone/RuCl_3_ [35], KMnO_4_/OsO_4_ [36], and KMnO_4_/18-crown-6 [37]). However, it was not possible to achieve satisfactory yields of the target carboxylic acid in any case due to the occurrence of adverse reactions involving chlorin oxidation.

In this regard, we implemented a two-step synthesis to obtain an intermediate chlorin 4 with a formyl group, which was obtained via Lemieux–Johnson oxidation [38] by the action of a catalytic amount of osmium tetroxide and an excess of sodium metaperiodate. The resulting aldehyde 4 was subjected to the Pinnick oxidation [39] using sodium chlorite and DMSO as an HClO [40] scavenger, instead of the traditional 2-methyl-2-butene used for this purpose.

To incorporate 6-hydrazinylnicotinic acid into the chlorin molecule, ethylenediamine was used as a linker. The amidation reaction of chlorin 5 with Boc-ethylenediamine was performed using HBTU to obtain chlorin 6 with a high yield. We demonstrated that this activating agent results in a faster reaction compared to using EDC and NHS. The reaction time was reduced from 2 h to 30–40 min. Characteristic signals of protons from methylene groups, amide groups, and a *tert*-butoxycarbonyl protecting group were observed in the ^1^H NMR spectrum (Figure S3).

The transformations of substituents in the pyrrole ring A of the chlorin macrocycle were accompanied by significant changes in the spectral properties of the chlorin molecule. Thus, when the vinyl group of chlorin was oxidized to the formyl group, a bathochromic shift of the absorption maximum in the electron spectrum was observed, with a shift of 30 nm to 698 nm and the subsequent oxidation to carboxylic acid 5 was accompanied by a hypsochromic shift of the absorption maximum by 10 nm and another 15 nm during amidation (Figure S7). These phenomena are associated with a change in the electron density distribution in the aromatic system of the chlorin molecule and allowed us to evaluate the progress of the reaction spectrophotometrically.

In the subsequent phase of the synthesis, 6-hydrazinylnicotinic acid was introduced into the structure of protected amine 6, which was treated with trifluoroacetic acid to remove the *tert*-butoxycarbonyl protective group, after which, Boc-HYNIC 2 (Scheme 1) was introduced into the amidation reaction using the activated ester method. In the ^1^H NMR spectrum of the synthesized substance, the signals of the aromatic protons were detected in the low field range. When analyzing the reaction product using high-resolution mass spectrometry, a molecular ion was detected that corresponds to the structure of compound 7 (Figure S5). This substance did not dissolve in water, which significantly hindered its labeling with the ^99m^Tc isotope and conducting biological studies. To increase the hydrophilicity, a taurine residue was introduced into the structure of this chlorin through selective hydrolysis of the ester group of propionic residue at position 17 of the chlorin macrocycle followed by amidation of a free carboxyl group and obtaining water-soluble chlorin 8. Such selectivity of modification of one of the three ester groups is associated with increased reactivity of methyl ester of the propionic acid residue at position 17 compared to aromatic ester groups located at positions 13 and 15 of the macrocycle.

The final step in the process of obtaining the target compound was the removal of the *tert*-butoxycarbonyl group from the hydrazine moiety. The use of trifluoroacetic acid for this purpose resulted in the formation of unwanted trifluoroacetamides, which is consistent with the findings of other researchers [41]; at the same time, attempts to remove the trifluoroacetyl group under conditions of alkaline hydrolysis did not result in the production of the desired product. For this reason, we used an alternative approach, which included heating derivative 8 to 120 °C in a 0.1 M hydrochloric acid solution in an autoclave. In early experiments, using this method, oxidation of the hydrazinyl fragment to diazenyl was observed. This is apparently due to the presence of residual amounts of oxygen in the reaction mixture. By carrying out this reaction in an inert gas atmosphere, we completely solved this problem and were able to obtain the desired compound HYNIC-Chl with quantitative yield, which was confirmed by HPLC-MS (Figure S6).

### 2.2. Radiolabeling of HYNIC-Chl

^99m^Tc labeling of HYNIC-Chl was performed with tricine and EDDA to form the [^99m^Tc]Tc-HYNIC complex. The effect of incubation temperature and incubation time on the radiochemical yield of [^99m^Tc]Tc-HYNIC-Chl radiolabeling was studied (Table 1). At an incubation temperature of 60 °C, the RCY was 66.0 ± 0.7% after 15 min of incubation and reached a maximum value of 71.7 ± 0.6% for this temperature regime when the reaction time was increased to 60 min.

Increasing the incubation temperature to 85 °C for 15 min significantly (*p* < 0.05) improved the RCY from 66.0 ± 0.7% to 82.3 ± 0.3%. However, as the incubation time was prolonged to 30 and 60 min, the RCY experienced a decline to 74.5 ± 0.4% and 69.7 ± 0.5%, respectively. Incubation for 60 min at 85 °C also produced a flocculent precipitate in the solution which was easily disrupted by vortexing. Examining the highest incubation temperature, a decline in RCY to 68.8 ± 2.2% was detected after incubation at 95 °C for 15 min, which was significantly (*p* < 0.05) lower than the RCY after incubation at 85 °C for 15 min. When the incubation time at 95 °C was prolonged to 30 min, the RCY decreased significantly (*p* < 0.05) compared to the incubation time of 15 min. The formation of a flaky precipitate was also observed.

Based on the results of the radiolabel optimization study, an incubation time of 85 °C and incubation temperature of 15 min were determined to be the optimal conditions (Figures S8 and S9).

The RHT was in the range of 0.1−1.0% under incubation conditions at 60 °C and 85 °C for 15, 30, and 60 min (Figure S9). However, an increase in the RHT level to 3.61 ± 0.44% was observed when incubated at 95 °C for 15 min. Curiously, when the incubation time was increased to 30 min, the level of the radiocolloid content was less than 1%. The RHT after purification was less than 1% for all labeling conditions tested.

The in vitro stability test showed that the [^99m^Tc]Tc-HYNIC-Chl was stable for 2 and 4 after the labeling procedure. The ^99m^Tc-labeled HYNIC-Chl demonstrated 1−2% release of activity after 2 h and 3–4% after 4 h of incubation at room temperature in a light-protected place (Figure S11).

To evaluate the lipophilicity of [^99m^Tc]Tc-HYNIC-Chl, the water–n-octanol distribution coefficient was determined. The distribution coefficient was 1.24 ± 0.03, indicating the high lipophilic character of the complex.

### 2.3. In Vitro Studies/In Vitro Binding and Cellular Uptake Experiments

The in vitro cell-binding test for [^99m^Tc]Tc-HYNIC-Chl was performed using SKOV-3 (ovarian adenocarcinoma) and A-431 (epidermoid carcinoma) cell lines to assess the ability of chlorin to bind and accumulate in tumor cells. Three time points (1, 4, and 24 h) and the same concentration (100 nM) of ^99m^Tc-labeled HYNIC-Chl were used to evaluate the effect of incubation time on the rate of cellular association.

An in vitro cell-binding test using SKOV-3 cells was conducted for [99mTc]Tc-HYNIC-Chl. The test involved saturation studies with varying concentrations of the chlorin, specifically 10 nM, 100 nM, and 1000 nM. No significant differences were observed in the percentage of cell-associated activity across any of the concentrations tested. However, there was a slight tendency for cell-associated activity to decrease at the 10 nM concentration, although this finding was not statistically significant compared to the other concentrations. Notably, even at the highest concentration of 1000 nM, no blocking effect was detected. Consequently, the test involving the blocking of tumor cell receptors with excess studied substance was deemed unnecessary.

**Figure 1:**
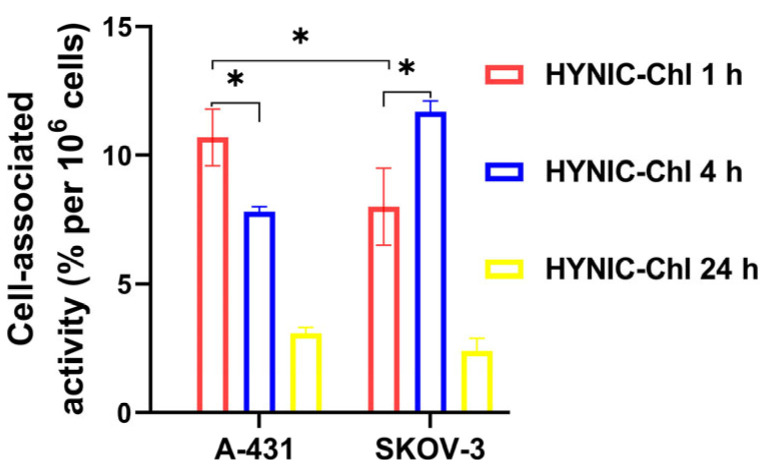
Cell-binding test of [^99m^Tc]Tc-HYNIC-Chl to A-431 and SKOV-3 cell lines. The final concentration of ^99m^Tc-labeled HYNIC-Chl was 100 nM. Symbol «*» marks a significant difference between groups (*p* < 0.05, unpaired *t*-test).

Results from the assessment of the cell-binding test for [^99m^Tc]Tc-HYNIC-Chl are shown in Figure 1. ^99m^Tc-labeled HYNIC-Chl tended to accumulate in tumor cells. For the A-431 cell line, the maximum accumulation detected was 10.7 ± 1.1% after 1 h of incubation and was reduced significantly (*p* < 0.05, unpaired *t*-test) to 7.8 ± 0.2% after 4 h of incubation. For the SKOV-3 cell line, the accumulation was 8.0 ± 1.5% after 1 h of incubation, and the maximum accumulation detected was 11.7 ± 0.4% after 4 h of incubation. Further, cellular retention decreased over time for both cell lines and was 3.1 ± 0.2% for SKOV-3 cells and 2.4 ± 0.5% for A-431 cells (*p* > 0.05, unpaired *t*-test) after 24 h of incubation. To determine the non-specific binding of chlorin to the surface of tumor cells and other sites, we incubated dead tumor cells and inhibited internalization in control dishes, showing 2% non-specific binding.

### 2.4. Biodistribution Study

**Figure 2:**
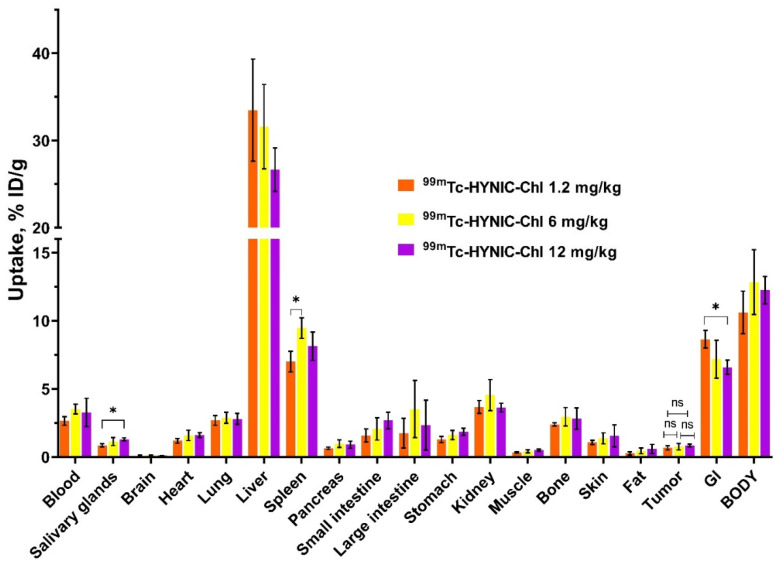
The dose-dependent biodistribution of [^99m^Tc]Tc-HYNIC-Chl (injected doses of 1.2 mg/kg, 6 mg/kg, and 12 mg/kg) in Nu/j mice bearing A-431 (epidermoid cancer tumor) xenografts at 2 h post injection (pi). Data are presented as mean %ID/g ± SD for four animals. Data for the rest of the gastrointestinal tract with contents (GI tract) and the rest of the body are presented as %ID per whole sample. The symbol «*» marks a significant difference between groups (*p* < 0.05, one-way ANOVA), and the symbol «ns» marks an insignificant difference between groups (*p* > 0.05, one-way ANOVA).

The results of the dose-dependent biodistribution of [^99m^Tc]Tc-HYNIC-Chl 2 h after injection into Nu/j mice bearing A-431 xenografts are presented in Figure 2 and Table S1. Doses of 6 mg/kg and 12 mg/kg were considered to be close to the expected therapeutic doses, and 1.2 mg/kg as a dose less than required for therapy. The biodistribution pattern showed common trends for all doses studied of 1.2 mg/kg, 6 mg/kg, and 12 mg/kg 2 h after injection, except for the uptake levels in several healthy organs. Blood retention was relatively high (below 3.5%ID/g) at all doses tested 2 h after injection. Tumor uptake values were 0.7 ± 0.2%ID/g, 0.8 ± 0.2%ID/g, and 0.8 ± 0.1%ID/g for the 1.2 mg/kg, 6 mg/kg, and 12 mg/kg doses, respectively. There was no significant difference between tumor uptakes at any doses (*p* > 0.05).

The highest radioactivity accumulation of ^99m^Tc-labeled HYNIC-Chl was observed in the liver and was greater than 25%ID/g at all doses studied (*p* > 0.05). Uptake in the stomach, small intestine, and large intestine was negligible at all doses tested (*p* > 0.05). Nevertheless, relatively high uptake was also noted in the rest of the gastrointestinal tract with contents at all doses tested (*p* > 0.05). Renal retention was within a reasonable range from 3%ID/g to 5%ID/g. This indicates that renal excretion does not play a significant role in the elimination of the tested chlorin and that it is dominated by excretion via the hepatobiliary system.

The substantial differences in uptake activity between the doses investigated were observed in the spleen, where the injected dose of 1.2 mg/kg provided reduced retention (7.0 ± 0.8%ID/g) compared to the injected dose of 6 mg/kg (9.4 ± 0.8%ID/g) (*p* < 0.05, one-way ANOVA). Moreover, the accumulation of ^99m^Tc-labeled HYNIC-Chl in the rest of the gastrointestinal tract with contents was significantly reduced at the 12 mg/kg dose compared to the lower 1.2 mg/kg dose (*p* < 0.05, one-way ANOVA).

The low level of radioactivity uptake in the salivary glands and stomach indicates that the [^99m^Tc]Tc-HYNIC-Chl complex is stable in vivo. In addition, salivary gland accumulation was slightly but significantly (*p* < 0.05) lower at the 1.2 mg/kg dose compared to the 12 mg/kg dose, 0.9 ± 0.1%ID/g and 1.3 ± 0.1%ID/g, respectively.

**Figure 3:**
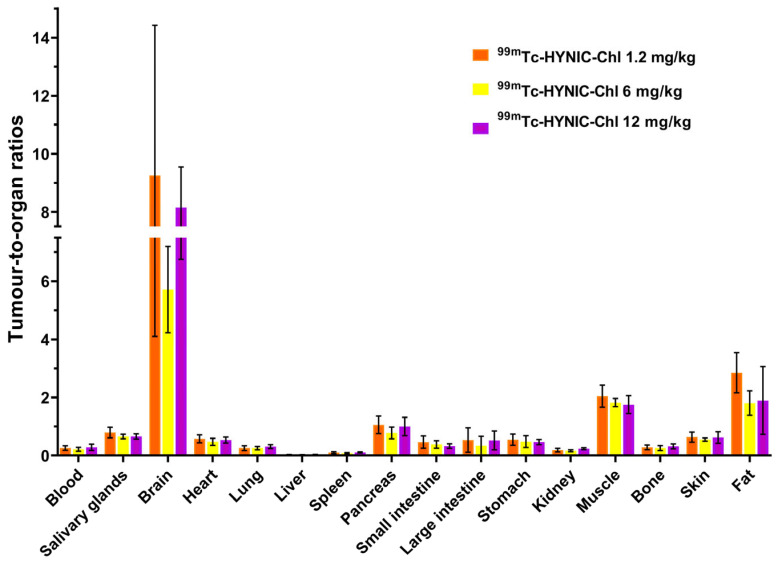
Dose-dependent tumor-to-organ ratios of [^99m^Tc]Tc-HYNIC-Chl (injected doses of 1.2 mg/kg, 6 mg/kg, and 12 mg/kg) in Nu/j mice bearing A-431 (epidermoid cancer tumor) xenografts at 2 h post injection (pi). Data are presented as mean %ID/g ± SD for four animals. Data for the rest of the gastrointestinal tract with contents (GI tract) and the rest of the body are presented as %ID per whole sample.

Based on the biodistribution data, tumor-to-organ ratios were calculated and are presented in Figure 3 and Table S2. The ratio of tumor-to-liver is critically low for all doses studied. The highest values were determined for tumor-to-brain, tumor-to-muscle, and tumor-to-fat ratios.

## 3. Discussion

As a result of chemical synthesis, we obtained the water-soluble derivative of chlorophyll A containing HYNIC residue in its structure. Among the BFCA-s, HYNIC was selected based on the efficiency of labeling and the ability to impart the hydrophilic character to the ^99m^Tc-labeled complex, which can therefore lead to a more favorable pharmacokinetic behavior in vivo concerning non-target accumulation in healthy organs. Varying the parameters of incubation time and temperature favored the high radiochemical yield of the complex. The obtained ^99m^Tc-labeled complex of chlorin is stable in vitro. However, a high LogD value was determined. The highly lipophilic nature of ^99m^Tc-labeled HYNIC-Chl raised concerns regarding subsequent biodistribution in vivo.

In vitro evaluation of [^99m^Tc]Tc-HYNIC-Chl showed that labeled chlorin successfully accumulated in both tumor cell lines tested. Herewith, maximum cellular accumulation for the A-431 cell line was detected after 1 h of incubation, and for the SKOV-3 cell line after 4 h of incubation. Regarding the later time point, cellular retention decreased significantly for both cell lines after 24 h of incubation. According to a proposed mechanism, the tested chlorin is internalized by the cell and undergoes cellular metabolism. The lipophilic radiometabolites of the HYNIC-chlorin complex can quickly leak through the cell membrane. This behavior of the radioactively labeled compound is characteristic of a non-residualizing label. After 24 h of incubation, the amount of metabolized chlorin molecules increased, resulting in their leakage from the cells as radiocatabolites. The variation in the level of cell-associated activity between different cell lines may be attributed to their differing proliferation rates. The A-431 cell line exhibits a higher proliferation rate compared to SKOV-3, leading to faster metabolic processes. Consequently, the test compound is metabolized more rapidly in A-431 cells. In the control group, internalization was slowed at 4 °C to determine the level of nonspecific binding of tested chlorin. The level of nonspecific binding was less than 2%, indicating a tumor-specific mechanism of chlorin accumulation.

Photosensitizers, particularly chlorin e_6_, are used in medical practice at relatively high doses. However, for diagnostic purposes, a lower dose of these agents can be employed compared to their therapeutic use. It is beneficial to select the lowest possible dose for the diagnostic agent to achieve high-contrast visualization of tumor uptake. Nonetheless, variations in the injected doses can significantly influence the substance’s biodistribution across organs and tissues and the extent of tumor uptake. Therefore, this study aimed to investigate the effects of different injected doses on biodistribution in vivo. The doses examined in this study included 6 mg/kg and 12 mg/kg, which are close to expected therapeutic levels, as well as 1.2 mg/kg, which is below the amount typically necessary for therapy. The dose-dependent biodistribution of [^99m^Tc]Tc-HYNIC-Chl showed common features at all doses studied, with minor exceptions in certain healthy organs. Biodistribution results show relatively high retention in the blood. This in vivo behavior is characteristic of chlorins due to binding to low-density lipoproteins, blood proteins, or other mechanisms, and was maintained for the complex of the tested chlorin with the [^99m^Tc]Tc-HYNIC core. The studied ^99m^Tc-labeled HYNIC-Chl tended to accumulate in the epidermoid carcinoma tumor. Notably, the accumulation in the tumor was not changed at the different doses tested.

The increased hepatic uptake is an unexpected feature for an HYNIC-containing chlorin. The [^99m^Tc]Tc-HYNIC core in combination with the hydrophilic coligands tricine and EDDA is hydrophilic and usually characterized by excretion via the kidneys. In general, the nature of the coligand used for labeling ^99m^Tc via HYNIC has a significant impact on the stability, lipophilicity, and biodistribution of the complex in vivo, mainly on binding to blood proteins, blood clearance rate, and liver uptake. Among the coligands used in HYNIC chemistry, it is EDDA and tricine that represent the best candidates for optimal in vivo biodistribution [42,43]. An alternative strategy could be a ternary complex using tricine/pyridine since literature data suggest that stabilization of tricine with pyridines reduces liver uptake [42].

In a study by Mohini Guleria et al., the investigated porphyrin [^99m^Tc]Tc-HYNIC-porphyrin also showed non-target uptake in the liver. The results of this study support the hypothesis that not only lipophilicity/hydrophobicity, but also other characteristics such as charge, size, and nature of functional groups involved in the ^99m^Tc coordination sphere, can greatly affect the uptake in vivo [13].

The retention of activity in the stomach and intestinal walls was not increased, but high levels of activity were found in the gastrointestinal contents. Considering the high accumulation in the liver and gastrointestinal contents, it can be concluded that the tested ^99m^Tc-labeled HYNIC-Chl is hepatobiliary excreted.

Significant differences among the doses studied were observed only in spleen and GI contents. The minimal injected dose of 1.2 mg/kg provided reduced retention in the spleen. In contrast, the highest dose of 12 mg/kg resulted in decreased accumulation in the gastrointestinal contents.

## 4. Materials and Methods

### 4.1. Synthesis of HYNIC-Chl

All the chemicals were obtained from commercial sources (Merck KGaA, Darmstadt, Germany; Acros Organics—part of Thermo Fischer Scientific, Waltham, MA, USA; Shanghai Makclin Biochemical Co., Ltd., Shanghai, China) and were used without further purification. Organic solvents were purchased from the CHIMMED company (Moscow, Russia), distilled, and dried using standard procedures. Column chromatography was performed on silica gel (Merck, Kieselgel 60, 40−63 μm). 6-(2-(*tert*-butoxycarbonyl)hydrazinyl)nicotinic acid (**2**) [7] and chlorin *p_6_* trimethyl ester (**3**) [44] were prepared according to reported procedures. All deuterated solvents were purchased from SOLVEX LLC (Moscow, Russia). NMR spectra were obtained on a Bruker DPX300 spectrometer (Bruker Corporation, Billerica, MA, USA) using CDCl_3_ and DMSO-d_6_ as solvents. Residual solvent was used as the reference standard for spectra calibrating. Electronic absorption spectra were obtained using a Shimadzu UV-1800 spectrophotometer (Shimadzu Corporation, Kyoto, Japan) in a 10 mm thick quartz cell.

Samples were analyzed on a Dionex UltiMate RS 3000 liquid chromatographic system (Thermo Scientific, Waltham, MA, USA) coupled with a Q-Exactive highresolution hybrid mass spectrometer (Thermo Scientific, Waltham, MA, USA). Separation of the sample components was performed on a pyramid reverse phase column with a C-18 bonded phase. Column length 75 mm, inner diameter 2 mm, and sorbent particle diameter 3 μm, manufactured by Macherey-Nagel, Düren, Germany (Cat. No. 37669012). Milli-Q deionized water (18.2 cm^−1^) was used as mobile phase (MP) component A. As component B of the mobile phase, we used isopropyl alcohol of the brand for HPLC “Carlo Erba”, France (Cat. No. UN 1219). Methyl alcohol for HPLC “Fisher Chemical”, Taiwan (Cat No. 1923759) was used as a wash solution for the autosampler. Flow rate PF is 0.400 mL. The elution mode is gradient. Change in the relative content of PF B (%) during the analysis: 0.00–1.00 min (5%); 1.00–12.00 min (95%); 12.00–18.00 min (95%); 18.00–18.01 min (5%); and 18.01–20.00 min (5%). The temperature of the column thermostat is 40 °C. Analysis time is 20.00 min. The volume of the aliquot applied to the column is 3.00 μL. The detection of compounds was carried out in the mode of detecting positively charged ions during heated electrospray ionization (HESI). Atomizing gas consumption (N2) 45 c.u.; auxiliary gas consumption 25 c.u., drying gas consumption 5 c.u. The voltage on the spraying capillary is 4.0 kV. The temperature of the spray capillary is 200 °C. The temperature of the inlet capillary is 350 °C. The voltage on the S lens is 50 c.u. Range of registration of values *m*/*z* 350–2200 relative units. Resolution 60,000 relative units.

#### 4.1.1. 3-Devinyl-3-formylchlorin p_6_ trimethyl ester (**4**)

Chlorin *p*_6_ trimethyl ester (**3**) (396 mg, 0.634 mmol) was dissolved in THF (20 mL). The resulting solution was cooled to 5 °C and a solution of sodium metaperiodate (543 mg, 2.539 mmol) in distilled water (6.7 mL) and OsO_4_ solution in CH_2_Cl_2_ with a concentration of 30.5 mg/mL (365 µL, 0.044 mmol) were added. The reaction mixture was vigorously stirred for 2 h at 25 °C, after which a solution of Na_2_S_2_O_3_∙5H_2_O (786 mg, 3.167 mmol) in water (4 mL) was added and the resulting mixture was stirred for 15 min. The reaction mixture was extracted with CH_2_Cl_2_ (20 mL), washed with water (2 × 20 mL), and then the organic phase was dried with anhydrous Na_2_SO_4_ and concentrated under reduced pressure. The target compound was isolated via column chromatography on a silica gel (n-hexane/ethyl acetate 1/1 *v*/*v*). Yield: 296 mg (74.5%).

UV/VIS (CH_2_Cl_2_) λ_max_, nm (ε, M^−1^ cm^−1^): 414 (41,000), 509 (3800), 544 (4400), 639 (3000), and 698 (21,300).

ESI-MS *m*/*z* calculated for C_35_H_38_N_4_O_7_ [M+H]^+^: 627.3, for C_36_H_40_N_4_O_9_ [M+HCOOH+H]^+^: 673.3. Found for [M+H]^+^: 627.2, for [M+HCOOH+H]^+^: 673.2.

^1^H NMR (300 MHz, CDCl_3_, δ, ppm): 11.41 (s, 1H, 3-COH), 10.12 (s, 1H, 10-H), 9.64 (s, 1H, 5-H), 8.82 (s, 1H, 20-H), 5.23–5.13 (m, 1H, 17-H), 4.46 (q, J = 6.7 Hz, 1H, 18-H), 4.26 (s, 3H, 13-CO_2_CH_3_), 4.21 (s, 3H, 15-CO_2_CH_3_), 3.76–3.61 (m, 8H, 8^1^-CH_2_, 17-CO_2_CH_3_, 12-CH_3_), 3.56 (s, 3H, 2-CH_3_), 3.21 (s, 3H, 7-CH_3_), 2.46 (m, 17^1^-CH_2_), 2.31–2.03 (m, 2H, 17^1^-CH_2_, 17^2^-CH_2_), 1.98–1.83 (m, 4H, 17^2^-CH_2_, 18-CH_3_), 1.66 (t, J = 7.6 Hz, 3H, 8^2^-CH_3_), −0.88 (br s, 1H, NH), and −1.32 (br s, 1H, NH).

#### 4.1.2. 3-Devinyl-3-carboxylchlorin p_6_ trimethyl ester (**5**)

Chlorin 4 (195 mg, 0.311 mmol) was dissolved in THF (7.7 mL). Water (770 µL), DMSO (1.105 mL, 15.563 mmol), and sulfamic acid (187 mg, 1.926 mmol) were added to the resulting solution. The solution of NaClO_2_ (168.8 mg, 1.867 mmol) in water (1 mL) was added dropwise over 2 h at 25 °C. Then Na_2_S_2_O_3_∙5H_2_O (386 mg, 1.555 mmol) was added to the reaction mixture and the resulting mixture was stirred for 15 min. The reaction mass was extracted with CH_2_Cl_2_ (15 mL), the organic phase was washed with water (2 × 10 mL), dried over anhydrous Na_2_SO_4_, and concentrated under reduced pressure. The target compound was isolated via column chromatography on silica gel (CH_2_Cl_2_/CH_3_OH 80/1 *v*/*v*). Yield: 96 mg (48.0%).

UV/VIS (CH_2_Cl_2_) λ_max_, nm (ε, M^−1^ cm^−1^): 406 (93,200), 504 (7900), 538 (7400), 630 (4900), and 689 (40,400).

ESI-MS *m*/*z* calculated for C_35_H_38_N_4_O_8_ [M+H]^+^: 625.3. Found: 643.3.

^1^H NMR (300 MHz, CDCl_3_, δ, ppm): 10.46 (s, 1H, 10-H), 9.68 (s, 1H, 5-H), 8.92 (s, 1H, 20-H), 5.21 (d, J = 8.5 Hz, 17-H), 4.50 (q, J = 7.1 Hz, 1H, 18-H), 4.27 (s, 3H, 13-CO_2_CH_3_), 4.22 (s, 3H, 15-CO_2_CH_3_), 3.83 (s, 3H, 17-CO_2_CH_3_), 3.74–3.62 (m, 5H, 8^1^-CH_2_, 12-CH_3_), 3.57 (s, 3H, 2-CH_3_), 3.27 (s, 3H, 7-CH_3_), 2.55–2.41 (m, 1H, 17^1^-CH_2_), 2.33–2.11 (m, 2H, 17^1^-CH_2_, 17^2^-CH_2_), 2.00–1.84 (m, 4H, 17^2^-CH_2_, 18-CH_3_), 1.69 (t, J = 7.6 Hz, 3H, 8^2^-CH_3_), and −1.30 (br s, 1H, NH).

#### 4.1.3. 3-Devinyl-3-((2-((tert-butoxycarbonyl)amino)ethyl)carbamoyl)chlorin p_6_ trimethyl ester (**6**)

Chlorin 5 (74.3 mg, 0.1156 mmol) was dissolved in DMF (1.820 mL). DIPEA (41 µL, 0.235 mmol) and HBTU (2-(1H-benzotriazol-1-yl)-1,1,3,3-tetramethyluronium hexafluorophosphate) (45.0 mg, 0.119 mmol) were added to the resulting solution. The reaction mixture was stirred for 35 min at 25 °C, after which *tert*-butyl (2-aminoethyl)carbamate (24 µL, 0.152 mmol) was added and the resulting solution was stirred for 5 min. The reaction mass was extracted with CH_2_Cl_2_ (10 mL), washed with water (2 × 10 mL), and the organic phase was dried over anhydrous Na_2_SO_4_ and concentrated under reduced pressure. The target compound was isolated via column chromatography on silica gel (CH_2_Cl_2_/CH_3_OH 80/1 *v*/*v*). Yield: 84.6 mg (93.2%).

UV/VIS (CH_2_Cl_2_) λ_max_, nm (ε, M^−1^ cm^−1^): 400 (123,300), 499 (10,100), 530 (5600), 618 (5200), and 674 (42,800).

^1^H NMR (300 MHz, CDCl_3_, δ, ppm): 9.75 (s, 1H, 10-H), 9.58 (s, 1H, 5-H), 8.68 (s, 1H, 20-H), 7.36 (br s, 1H, 3-CO-NH), 5.23 (br s, 1H, (CH_3_)_3_CO_2_C-NH-), 5.16 (d, J = 8.1 Hz, 1H, 17-H), 4.40 (q, J = 7.2 Hz, 1H, 18-H), 4.24 (s, 3H, 13-CO_2_CH_3_), 4.18 (s, 3H, 15-CO_2_CH_3_), 3.98–3.88 (m, 2H, 3-CO-NH-CH_2_), 3.68–3.46 (m, 13H, 3-CO-NH-CH_2_-CH_2_, 17-CO_2_CH_3_, 8^1^-CH_2_, 12-CH_3_, 2-CH_3_), 3.15 (s, 3H, 7-CH_3_), 2.49–2.33 (m, 1H, 17^1^-CH_2_), 2.27–2.02 (m, 2H, 17^1^-CH_2_, 17^2^-CH_2_), 1.92–1.75 (m, 4H, 17^2^-CH_2_, 18-CH_3_), 1.61 (t, J = 7.6 Hz, 3H, 8^2^-CH_3_), 1.37 (s, 9H, -CO_2_C(CH_3_)_3_), −1.05 (br s, 1H, pyrrole-NH), and −1.34 (br s, 1H, pyrrole-NH).

#### 4.1.4. 3-Devinyl-3-((2-(6-(2-(tert-butoxycarbonyl)hydrazinyl)nicotinamido)ethyl)carbamoyl)chlorin p_6_ trimethyl ester (**7**)

6-(2-(*tert*-Butoxycarbonyl)hydrazinyl)nicotinic acid (2) (94.6 mg, 0.374 mmol), DIPEA (135 µL, 0.775 mmol), and and HBTU (141.4 mg, 0.373 mmol) were dissolved in DMF (800 µL). The resulting mixture was stirred for 1 h at 25 °C. In a separate flask, chlorin 6 (196.1 mg, 0.250 mmol) was dissolved in a freshly prepared solution of CF_3_COOH in CH_2_Cl_2_ (1/1 *v*/*v*, 1.6 mL). The resulting solution was stirred for 10 min at 25 °C. The reaction mass was dissolved with CH_2_Cl_2_ (20 mL) and washed with a saturated aqueous solution of NaHCO_3_ (2 × 10 mL). The organic phase was dried with anhydrous Na_2_SO_4_ and concentrated under reduced pressure. The residue was dissolved in DMF (2.5 mL) and added to a solution of the activated ester of 2. The reaction mixture was stirred for 20 min at 25 °C, then extracted with CH_2_Cl_2_ (20 mL) and washed with water (2 × 10 mL). The organic phase was washed with anhydrous Na_2_SO_4_ and concentrated under reduced pressure. The target compound was isolated via column chromatography on silica gel (CH_2_Cl_2_/CH_3_OH 25/1 *v*/*v*). Yield: 144.7 mg (63.0%).

UV/VIS (CH_2_Cl_2_) λ_max_, nm (ε, M^−1^ cm^−1^): 260 (32,200), 400 (166,500), 499 (13,400), 530 (79,500), 619 (7100), and 675 (56,000).

ESI-HRMS *m*/*z* calculated for C_48_H_57_N_9_O_10_ [C_48_H_57_N_9_O_10_+H]^+^: 920.4307. Found: 920.4276.

^1^H NMR (300 MHz, CDCl_3_, δ, ppm): 9.34 (s, 1H), 9.24 (s, 1H), 8.58 (s, 1H), 8.41 (s, 1H), 7.63 (s, 1H), 7.53 (s, 2H), 6.54 (s, 1H), 6.23 (s, 1H), 6.12 (s, 1H)–10-H, 5-H, 20-H, HYNIC-Boc-H, 3-CO-NH-CH_2_CH_2_-NH, 5.19–5.10 (m, 1H, 17-H), 4.37 (q, J = 7.4 Hz, 1H, 18-H), 4.24 (s, 3H, 13-CO_2_CH_3_), 4.18 (s, 3H, 15-CO_2_CH_3_), 3.80–3.32 (m, 10H, 8^1^-CH_2_, 3-CO-NH-CH_2_-CH_2_, 17-CO_2_CH_3_, 12-CH_3_), 3.31–3.00 (m, 3H, 2-CH_3_, 3-CO-NH-CH_2_-CH_2_), 2.77 (s, 3H, 7-CH_3_), 2.47–2.34 (m, 1H, 17^1^-CH_2_), 2.28–2.01 (m, 2H, 17^1^-CH_2_, 17^2^-CH_2_), 1.84–1.75 (m, 4H, 17^2^-CH_2_, 18-CH_3_), 1.41–1.35 (m, 3H, 8^2^-CH_3_), 1.24 (s, 9H, -CO_2_C(CH_3_)_3_), and −1.50 (br s, 1H, pyrrole-NH).

#### 4.1.5. 3-Devinyl-3-((2-(6-(2-(tert-butoxycarbonyl)hydrazinyl)nicotinamido)ethyl)carbamoyl)-17^3^-((2-sulfoethyl)carbamoyl)chlorin p_6_ 13^1^,15^1^-dimethyl ester (**8**)

An aqueous solution of NaOH (2 M, 60 mL) was added to the solution of chlorin **7** (127.0 mg, 0.138 mmol) in THF (60 mL) while stirring. The resulting mixture was stirred at 25 °C for 12 h. The reaction mixture was extracted with CH_2_Cl_2_ (40 mL), washed with citrate buffer solution (pH = 4.00, 2 × 20 mL), dried with anhydrous Na_2_SO_4_, and concentrated under reduced pressure.

The residue was dissolved in DMF (2 mL). DIPEA (39 µL, 0.2239 mmol) and HBTU (42 mg, 0.111 mmol) were added to the solution. The resulting mixture was stirred for 20 min at 25 °C, after which taurine (33 mg, 0.264 mmol) was added. The resulting solution was stirred for 40 min at 25 °C, then was extracted with CH_2_Cl_2_ (20 mL) and washed with citrate buffer solution (pH = 4.00, 2 × 10 mL). The organic phase was dried over anhydrous Na_2_SO_4_ and concentrated under reduced pressure. The target compound was isolated via flash-chromatography on silica gel (CH_2_Cl_2_/CH_3_OH 10/1, then 5/1 *v*/*v*). Yield: 96.8 mg (69.2%).

UV/VIS (DMSO) λ_max_, nm (ε, M^−1^ cm^−1^): 269 (38,100), 400 (190,600), 498 (15,300), 528 (7500), 614 (7600), and 669 (61,000).

ESI-HRMS *m*/*z* calculated for C_49_H_60_N_10_O_12_ [C_49_H_60_N_10_O_12_+H]^+^: 1013.4191. Found: 1013.4171.

#### 4.1.6. 3-Devinyl-3-((2-(6-hydrazinylnicotinamido)ethyl)carbamoyl)-17^3^-((2-sulfoethyl)carbamoyl)chlorin p_6_ 13^1^,15^1^-dimethyl ester (**9**)

Chlorin 8 (12.2 mg, 0.012 mmol) was dissolved in degassed distilled water (6.1 mL) and the aqueous solution of HCl (1 M, 1.22 mL) was added to the resulting solution. The reaction mixture was stirred for 1 h at 120 °C in a sealed flask. After cooling to 25 °C, the resulting solution was concentrated under reduced pressure. Yield: 10.1 mg (92.2%).

ESI-HRMS *m*/*z* calculated for C_49_H_60_N_10_O_12_ [C_49_H_60_N_10_O_12_]^+^: 913.3667. Found: 913.3632.

### 4.2. Radiolabeling Optimization

For radiolabeling, to 250 μg of HYNIC-Chl (100 μL, 2.5 mg/mL dissolved in dimethyl sulfoxide (DMSO)/water; 1/1 (*v*/*v*)) was added tricine (10 mg, 100 μL, and 0.1 g/mL in PBS, Sigma-Aldrich, Germany), EDDA (5 mg,100 μL, 0.05 g/mL in 0.4 M NaOH, Fluka Chemika, Switzerland) tin (II) chloride dihydrate (50 µg, 25 µL, and 2 mg/mL in 0.1 M HCl, Fluka Chemika, Buchs, Switzerland), and 100 µL of ^99m^Tc-pertechnetate generator eluate (150–250 MBq), followed by vortexing. To investigate the optimal radiolabeling conditions, the mixture was incubated by varying the temperature and the reaction time. The effect of the reaction time was studied by applying time variations of 15 min, 30 min, or 60 min to the reaction process. Temperature variations of 60 °C, 85 °C, or 95 °C were used to study the effect of incubation temperature.

Once the optimum conditions for radiolabeling were established (or after establishing optimal conditions for radiolabeling), the [^99m^Tc]Tc-HYNIC-Chl was purified by using a C18 Sep-Pak cartridge (360 mg, Waters Corp, Milford, MA, USA). The cartridge was pre-conditioned by passing 10 mL of 95% ethanol solution, 10 mL of 50% ethanol solution, and 10 mL of Milli-Q water before the loading of the radiolabeled preparation. Free radiometal was eluted using 40 mL of Milli-Q water and subsequently, the ^99m^Tc-labeled HYNIC-Chl was eluted from the column using 2 mL of 95% ethanol solution.

Radiolabeling yield and radiochemical purity of [^99m^Tc]Tc-HYNIC-Chl were determined by instant thin-layer chromatography (radio-ITLC) (silica gel strips as a stationary phase and two mobile phases A: PBS and B: acetonitrile: water (1:1) were used). Radiolabeling yield and radiochemical purity of [^99m^Tc]Tc-HYNIC-Chl were determined by instant thin-layer chromatography (radio-ITLC) using silica gel strips as stationary phase and two mobile phases A: PBS and B: acetonitrile: water (1:1). In system A, ^99m^Tc-labeled HYNIC-Chl and the reduced hydrolyzed technetium colloid (RHT, [^99m^Tc]TcO_2_) stayed at the application point (*R*_f_  =  0.0), whereas free forms of radionuclide ions and ^99m^Tc-labeled complexes with tricine and EDDA migrate with the solvent front (*R*_f_  =  0.8–1.0). The RHT level was measured using system B (^99m^Tc colloid stayed at the application point, *R*_f_  =  0.0, other forms of ^99m^Tc ions and ^99m^Tc-labeled HYNIC-Chl, and *R*_f_  =  1.0). For the calculation of radiochemical purity, the percentage of RTH-associated activity (analysis using system B) was subtracted from the percentage of activity at the application point in system A.

The in vitro stability test of the radioactive complex was performed using the radio-iTLC method. The labeled samples (n = 2) were kept at room temperature in a light-protected place and after 2 h and 4 h incubation, the percentage of release radioactivity from the ^99m^Tc-labeled chlorin complex was measured using radio-ITLC.

The octanol–water distribution coefficient (Log D) was determined by adding 10 µL of ^99m^Tc-labeled HYNIC-Chl to a LoBind Eppendorf tube containing 500 µL of Milli-Q water and 500 µL of n-octanol. The tube was thoroughly vortexed for 3 min, then centrifuged for 5 min. Fractions of each phase (100 μL) were collected in a pre-weighed tube and their activity and weight were measured. The experiment was performed in triplicate. The LogD value was calculated per 100 μg of ^99m^Tc-labeled chlorin.

### 4.3. In Vitro Studies/In Vitro Binding and Cellular Uptake Experiments

In vitro characterization of [^99m^Tc]Tc-HYNIC-Chl complex was performed in SKOV-3 (ovarian adenocarcinoma) and A-431 (epidermoid carcinoma) cell lines purchased from the American Type Culture Collection (ATCC, LGC Promochem, Buros, Sweden).

To evaluate the level of cellular association, the cells were seeded one day prior to the experiments into Petri dishes (3.5 cm diameter, nine dishes for each tested cell line) with a density of 7 × 10^5^ cells/dish. The cells were incubated for 24 h at 37 °C, 5% CO_2_ until a monolayer culture was formed. On the day of the in vitro experiment, the culture medium was removed, and cells were washed with 1 mL PBS. Next, the solution containing ^99m^Tc-labeled chlorin was simultaneously added to each dish to a concentration of 100 nM. The dishes were incubated for 1/4/24 h (three dishes per time point for each cell line) at 37 °C, 5% CO_2_.

Two groups of control dishes were used as negative controls. In the first control group, three dishes received the same concentration of ^99m^Tc-labeled HYNIC-Chl (100 nM) and were incubated for 1 h at 4 °C to prevent internalization. This incubation aimed to assess the non-specific binding of chlorins to the surface of tumor cells. In the second control group, the same concentration of ^99m^Tc-labeled HYNIC-Chl (100 nM) was added to three dishes containing dead tumor cells, which were obtained by freezing without protective agents or by treatment with ethanol. After the required incubation period, cell medium was collected; the cells were harvested after trypsin treatment, followed by washing with PBS. The activity in fractions containing medium and cells was measured using a gamma-spectrometer with a NaI (TI) detector (2480 Wizard2, Perkin Elmer, Waltham, MA, USA), and the percentage of cell-associated activity was calculated.

### 4.4. In Vivo Studies

To establish epidermoid carcinoma xenograft, 10^7^ of A-431 cells in 100 µL of media were subcutaneously injected into hind legs of female Nu/J mice two weeks before the start of the experiment. At the time of the experiments, the average animal and xenograft weights were 25.4 ± 1.7 g and 0.41 ± 0.31 g, respectively. Mice were randomized into three groups. A group of four mice was used per data point.

To assess tumor uptake and study the effect of dose on the biodistribution, mice bearing A-431 xenografts were injected intravenously into the tail vein with 1.2 mg/kg (30 µg/mouse, 60 kBq), 6 mg/kg (150 µg/mouse, 60 kBq), and 12 mg/kg (300 µg/mouse, 60 kBq) of [^99m^Tc]Tc-HYNIC-Chl. The injected mass of chlorin (up to 30, 150, or 300 μg depending on the injected dose) was adjusted by a corresponding non-labeled HYNIC-Chl. The injected volume was adjusted with PBS to 100 µL/mouse.

Animals were euthanized under anesthesia by cervical dislocation. The organs were collected and weighed. The activity in the samples was measured using an automatic gamma-spectrometer with a NaI (TI) detector (2480 Wizard2, Perkin Elmer, Waltham, MA, USA), and the associated activity was calculated as the percentage of injected dose per gram of sample of the corresponding organ or tissue (%ID/g).

### 4.5. Statistical Analysis

Statistical analysis was performed using GraphPad Prism (version 8.0; GraphPad Software, Inc., La Jolla, CA, USA). In the in vitro studies, an unpaired two-tailed *t*-test was used to determine statistically significant differences between the two groups. In the in vivo studies, data were assessed either by one-way ANOVA with Tukey correction for multiple comparisons to determine significant differences between groups (*p* < 0.05). In the figures, «*» marks a statistically significant difference (*p* < 0.05, unpaired, two-tailed *t*-test), and «ns» marks a statistically insignificant difference (*p* > 0.05, unpaired, two-tailed *t*-test).

## 5. Conclusions

As a result of this work, we developed a new ligand for the coordination of ^99m^Tc, which consists of a fragment of 6-hydrazinylnicotinic acid and a natural chlorin. It was shown that, despite its high radiolabeling efficiency, this conjugate does not have high selectivity for accumulation in tumor tissue at any of the studied doses. This is likely due to its high lipophilicity. Currently, we are developing new water-soluble derivatives of chlorin with ^99m^Tc chelators, which have increased hydrophilicity. The goal is to find a lead compound, a prototype for theranostics, which will be used for combined radionuclide imaging and photodynamic therapy.

## Figures and Tables

**Table 1:** Radiochemical yield from radiolabeling optimization studies, isolated yield, radiochemical purity, and maximum molar activity of HYNIC-Chl radiolabeled with [^99m^Tc]TcO_4_^−^.

Temperature, °C	Radiochemical Yield *, %			Isolated Yield *, %	Radiochemical Purity *, %	Maximum Molar Activity, MBq/nmol
	Time, min					
	15	30	60			
60	66.0 ± 0.7	70.7 ± 0.5	71.7 ± 0.6	42.1	94.6 ± 0.1	0.55
85	82.3 ± 0.3	74.5 ± 0.4	69.7 ± 0.5	49.8	94.9 ± 0.5	0.62
95	68.8 ± 0.4	63.3 ± 0.9	40.1 ± 0.8	42.6	92.3 ± 1.5	0.42

* Radiochemical yield is determined by radio-iTLC analysis of the reaction mixture before purification. Isolated yield is defined as the percentage of activity measured in ethanol fraction after purification. Radiochemical purity is determined by radio-iTLC analysis of the purified [^99m^Tc]Tc-HYNIC-Chl (Figure S10). Experiments were performed in duplicates.

## Data Availability

The original contributions presented in the study are included in the article/Supplementary Materials, further inquiries can be directed to the corresponding authors.

## Supplementary Materials

The following supporting information can be downloaded at: https://www.mdpi.com/article/10.3390/molecules30010117/s1, Figures S1–S4: NMR spectra of compounds **4**–**7**; Figures S5–S6: Chromatograms and high-resolution mass spectra of HYNIC-containing compounds; Figure S7: UV-vis absorbance spectra of compounds **3**–**6**; Figures S8–S11: Radio-iTLC chromatograms; Tables S1–S2: Biodistribution data of ^99m^Tc-HYNIC-Chl.
