# Supplementary material for: Novel Chlorin with a HYNIC: Synthesis, 99mTc-Radiolabeling, and Initial Preclinical Evaluation
Source: Molecules. 2024 Dec 31;30(1):117. doi: 10.3390/molecules30010117 (PMC11721277; doi:10.3390/molecules30010117)
Supplement: Supplementary file 1 [file molecules-30-00117-s001.zip › molecules-3339587-supplementary.pdf]

Supporting information

Novel Chlorin with a HYNIC: Synthesis, ^{99m}Tc-Radiolabeling, and Initial Preclinical Evaluation

Alexander Popov [1,*], Nikita Suvorov [1], Mariia Larkina [2,3], Evgenii Plotnikov [2,3], Ruslan Varvashenya [2,3], Vitalina Bodenko [2,3], Gleb Yanovich [2,3], Petr Ostroverkhov [1], Maxim Usachev [1], Elena Filonenko [1,4], Mikhail Belousov [5], Mikhail Grin [1]

[1] M.V. Lomonosov Institute of Fine Chemical Technology, MIREA—Russian Technological University, 86 Vernadsky Av., 119571 Moscow, Russia; alexander.p.tmb@gmail.com (A.P.); suvorov.nv@gmail.com (N.S.); mrp_ost@mail.ru (P.O.); maximus021989@mail.ru (M.U.); elena.filonenko@list.ru (E.F.); michael_grin@mail.ru (M.G.);
[2] Science and Education Laboratory for Chemical and Pharmaceutical Research, Siberian State Medical University, 634050 Tomsk, Russia; bodenkovitalina@gmail.com (V.B.); sonne_gleb@mail.ru (G.Y.); mr.varvashenya@mail.ru (R.V.)
[3] Research Centrum for Oncotheranostics, Research School of Chemistry and Applied Biomedical Sciences, Tomsk Polytechnic University, 634050 Tomsk, Russia; marialarkina@mail.ru (M.S.); plotnikovev@tpu.ru (E.P.);
[4] P. A. Hertsen Moscow Oncology Research Center, 125284 Moscow, Russia;
[5] Department of Pharmaceutical Analysis, Siberian State Medical University, 634050 Tomsk, Russia; mvb63@mail.ru (M.B.);
* Correspondence: alexander.p.tmb@gmail.com (A.P.).

Contents

NMR spectra of compounds **4**-**7** 3

Chromatograms and high resolution mass spectra of HYNIC-containing compounds 7

UV-vis absorbance spectra of compounds **3**-**6** 9

Radio-iTLC chromatograms 10

Biodistribution data of ^{99m}**Tc-HYNIC-Chl** 14

NMR spectra of compounds 4-7

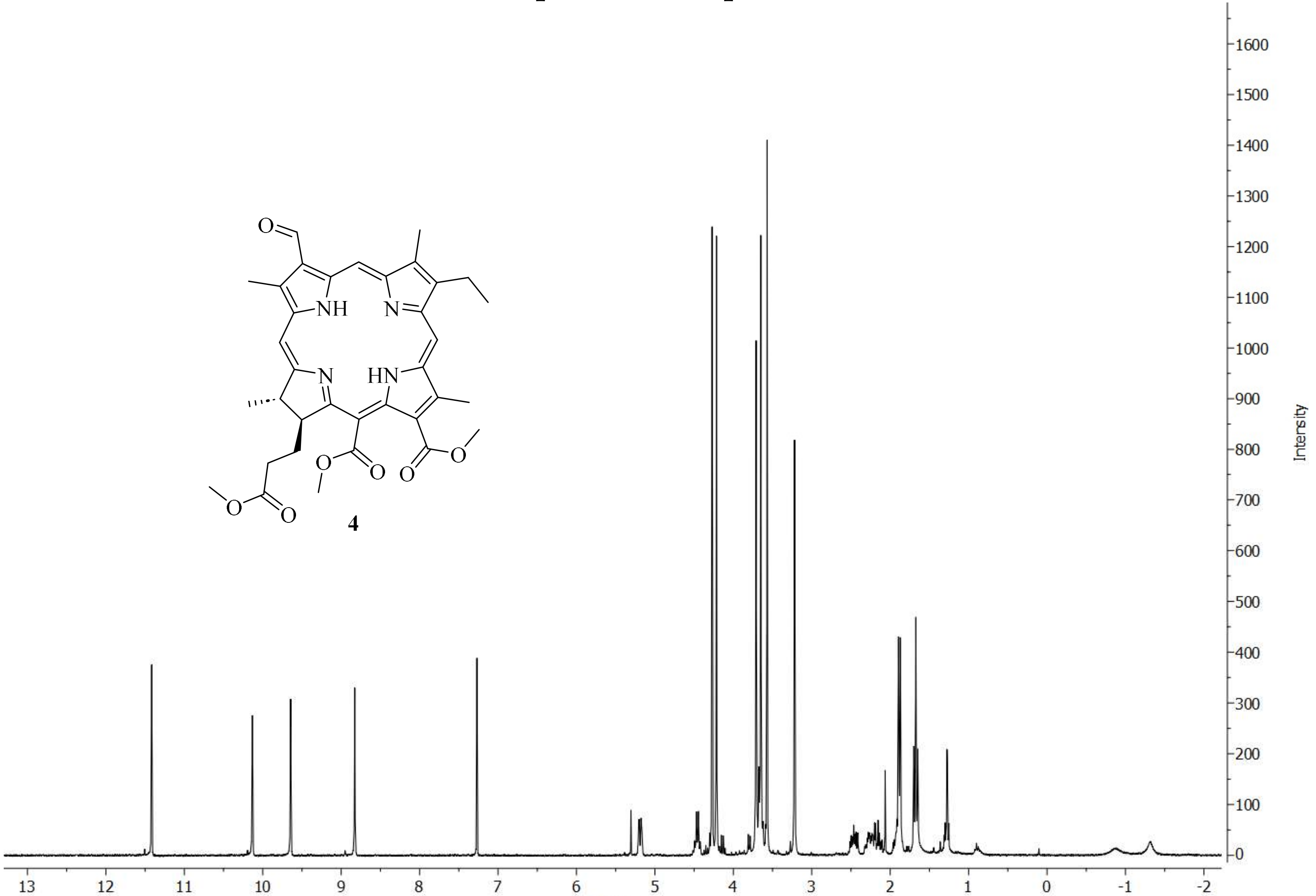

Figure S1: ^{1}H NMR spectrum of compound **4** (solvent: $CDCl_3$)

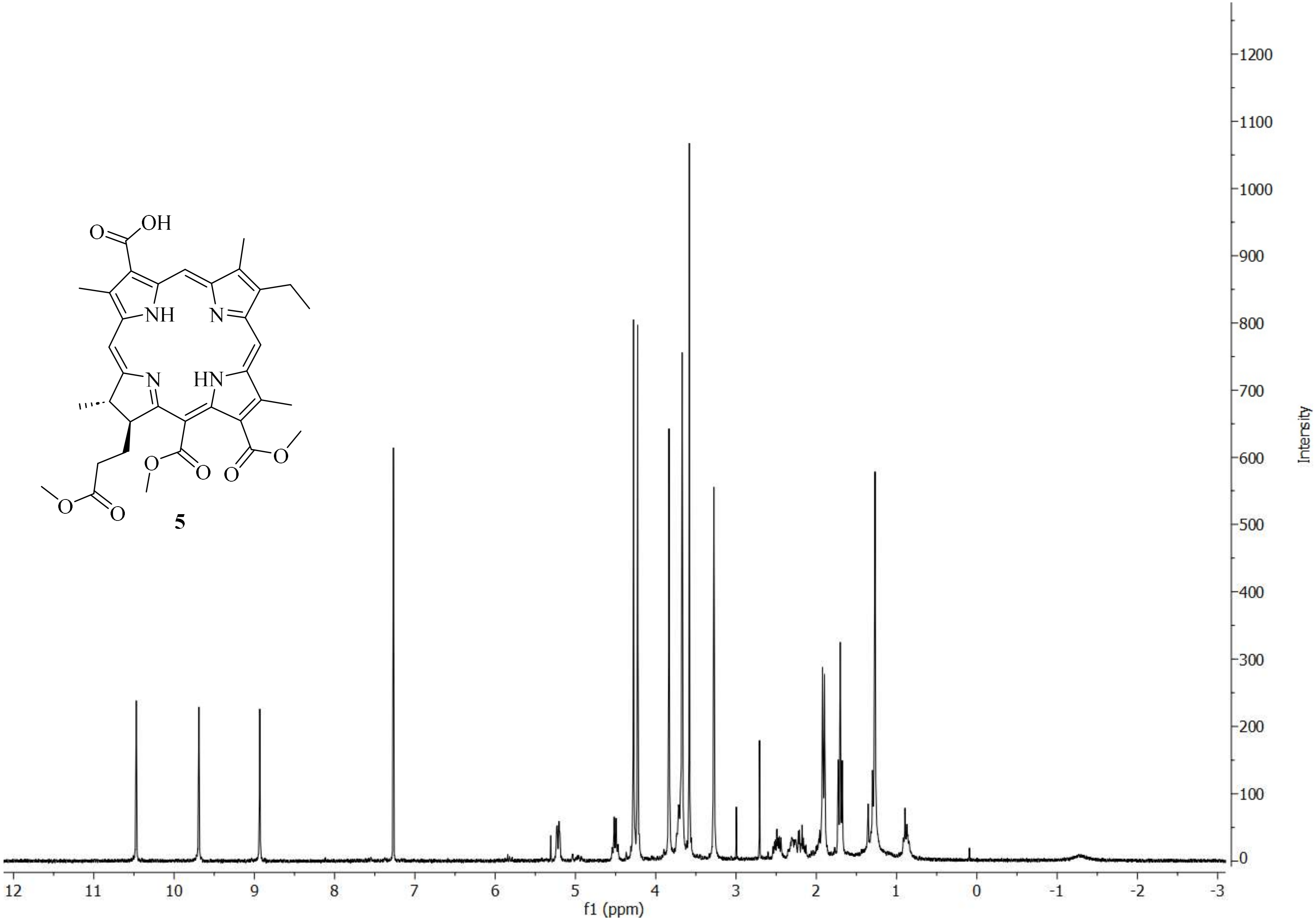

Figure S2: ^{1}H NMR spectrum of compound **5** (solvent: $CDCl_3$)

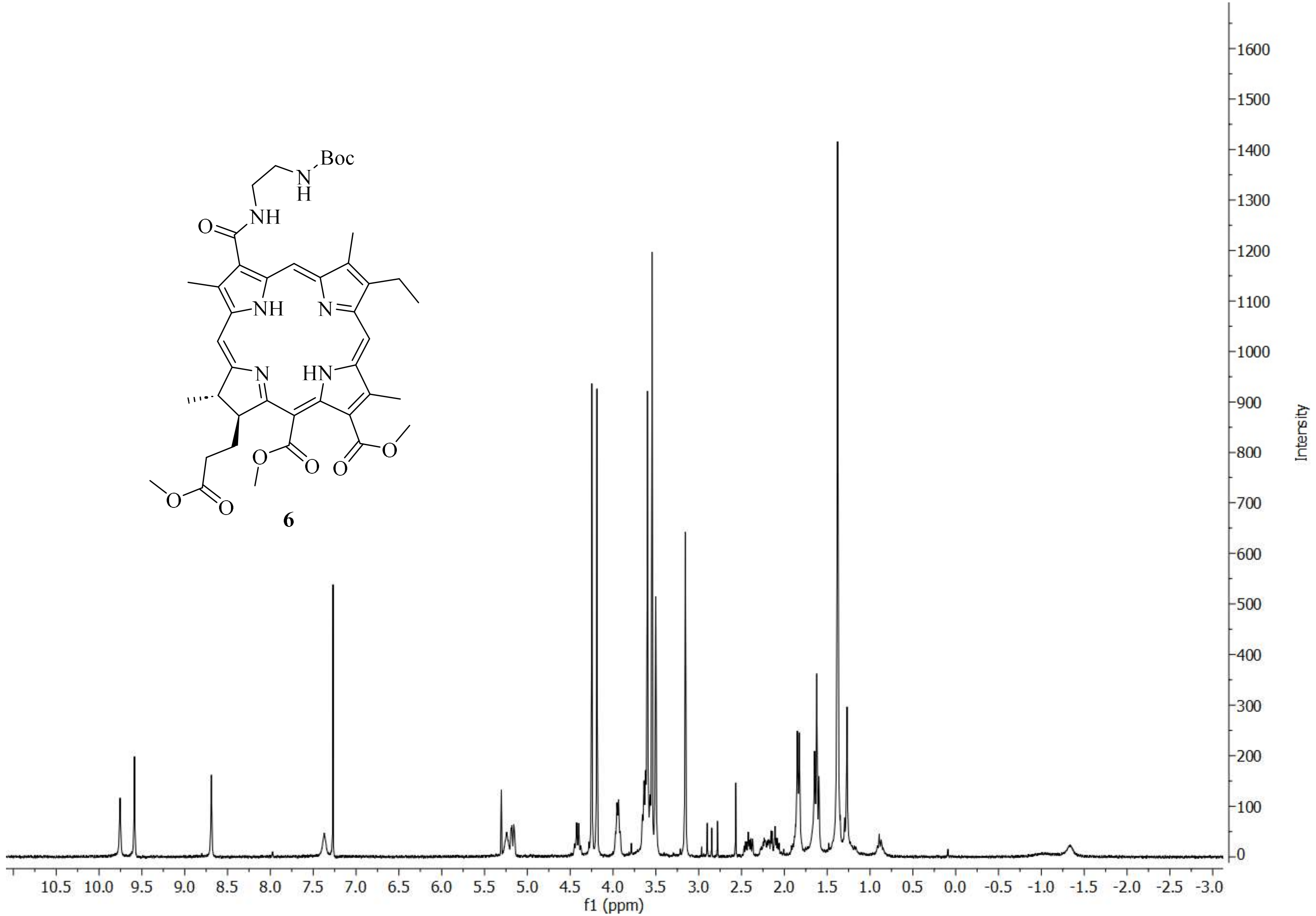

Figure S3: ^{1}H NMR spectrum of compound **6** (solvent: $CDCl_3$)

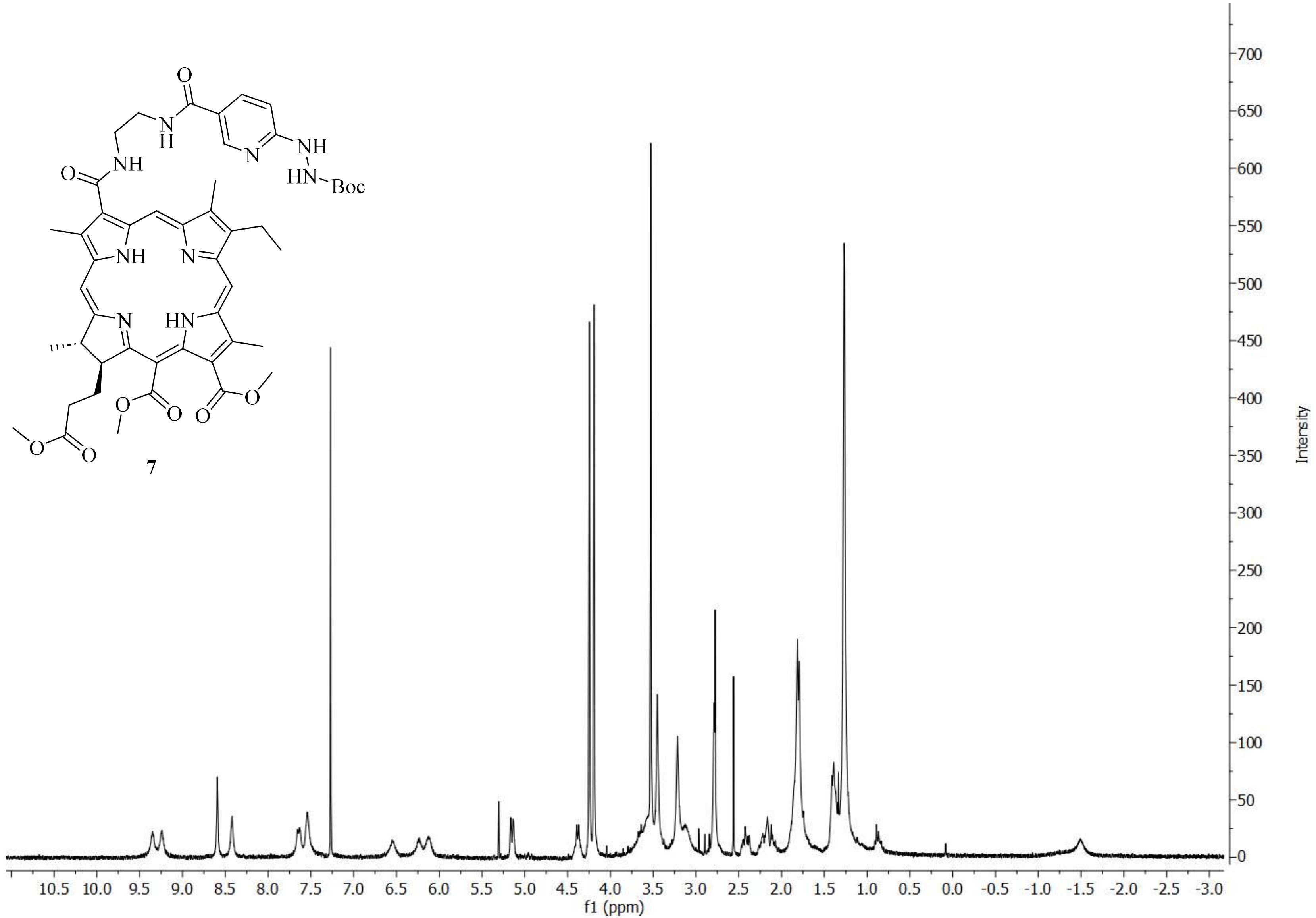

Figure S4: ^{1}H NMR spectrum of compound **7** (solvent: $CDCl_3$)

Chromatograms and high resolution mass spectra of HYNIC-containing compounds

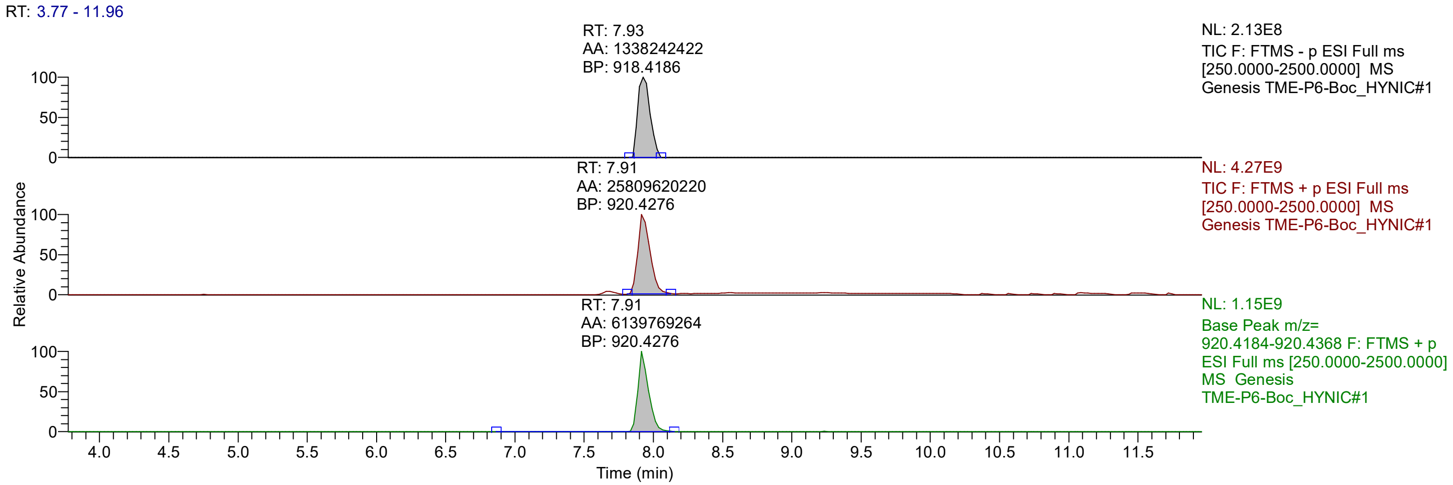

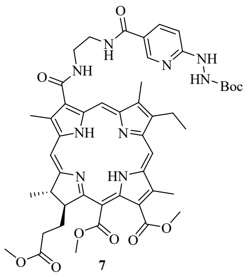

Chemical Formula: $C_{48}H_{57}N_9O_{10}$
Exact Mass: 919,4228

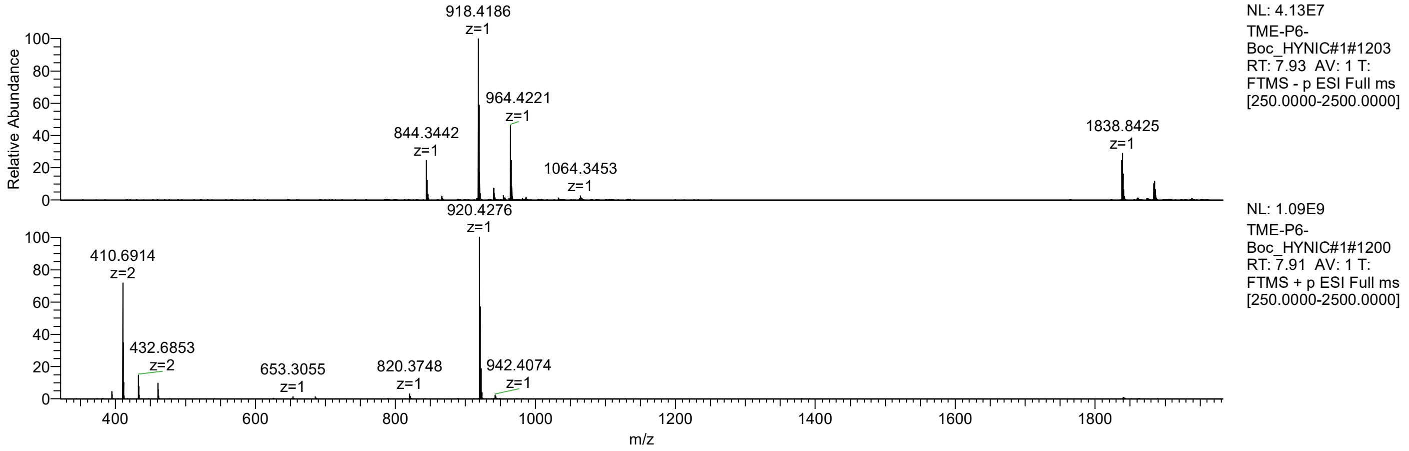

Figure S5: Chromatogram and high-resolution mass spectrum of compound **7**

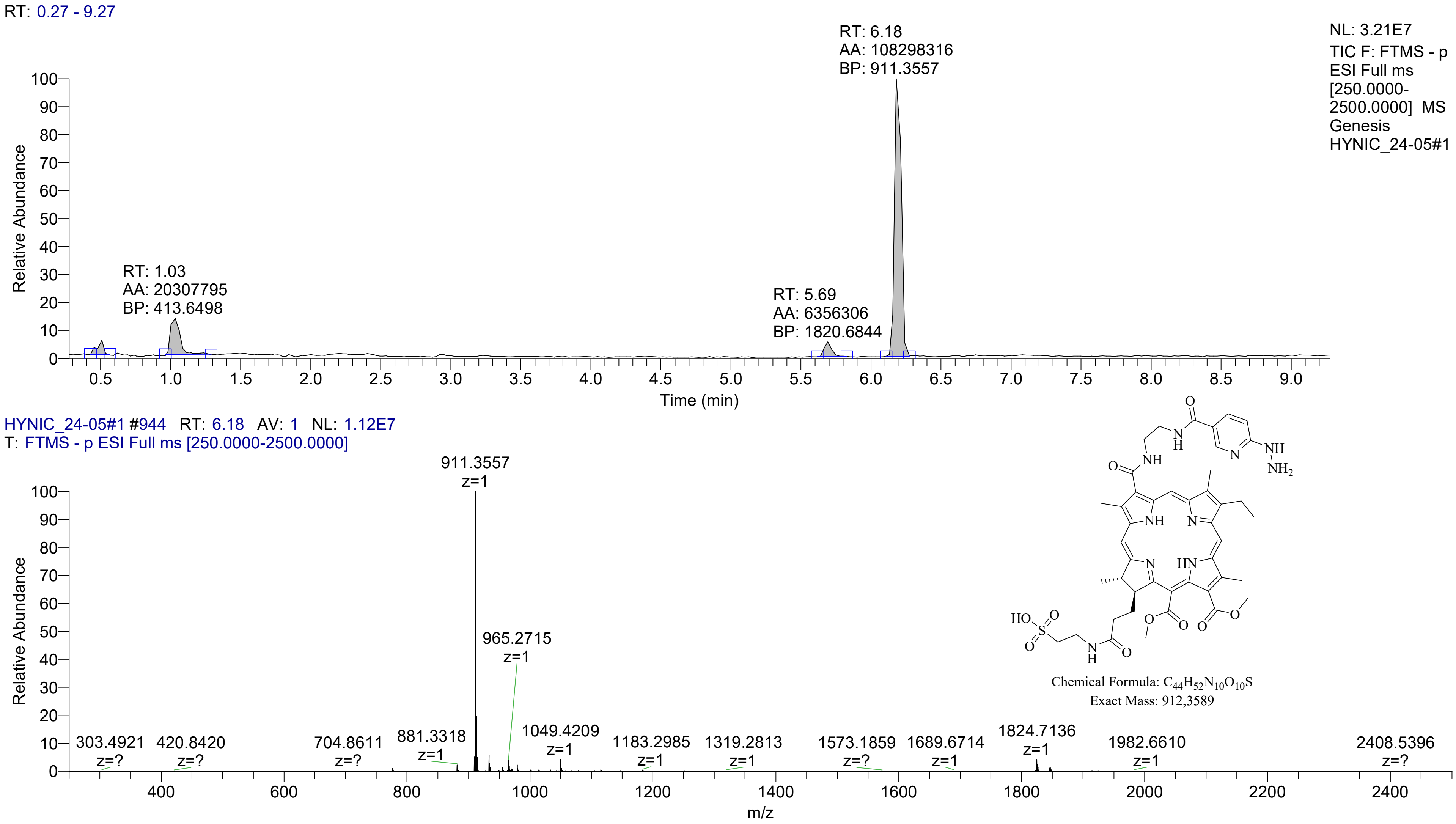

Figure S6: Chromatogram and high-resolution mass spectrum of **HYNIC-Chl**

UV-vis absorbance spectra of compounds 3-6

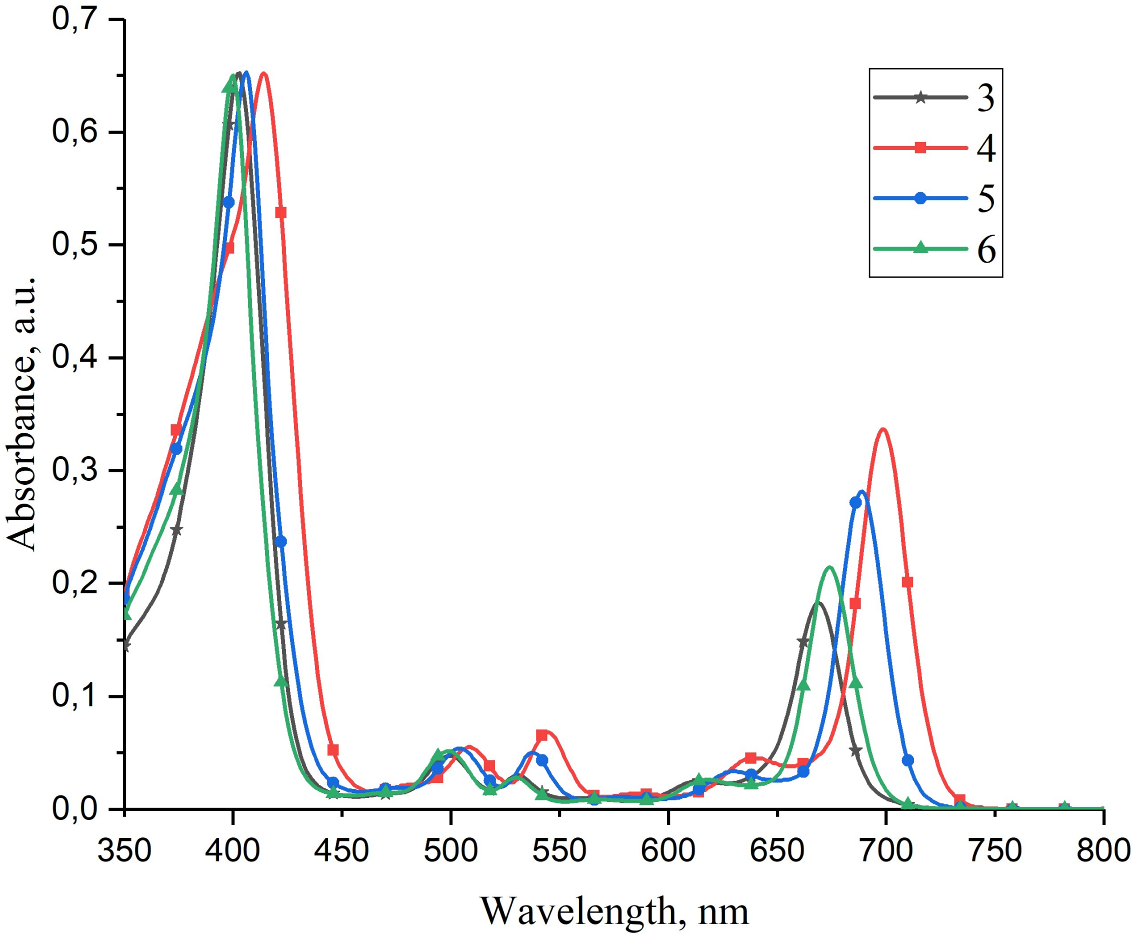

Figure S7: UV-vis absorbance spectra of compounds **3**-**6** in CH_2Cl_2

Radio-iTLC chromatograms

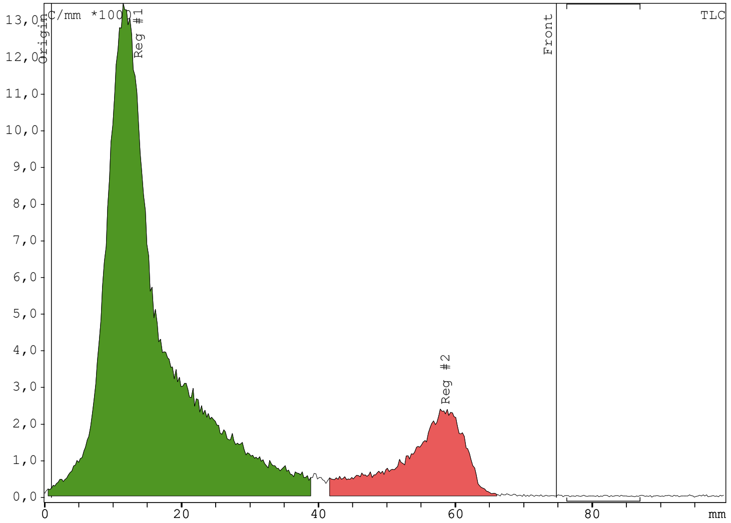

Figure S8: Radio-iTLC chromatogram of [^{99m}Tc]Tc-HYNIC-Chl yield after incubation at 85°C for 15 min (PBS as a mobile phase). The peak 1 corresponds to a mixture of [^{99m}Tc]Tc-HYNIC-Chl and [^{99m}Tc]TcO_2 impurity, the peak 2 corresponds to free $^{99m}TcO_4^-$ ions, [^{99m}Tc]Tc-EDDA and [^{99m}Tc]Tc-tricine.

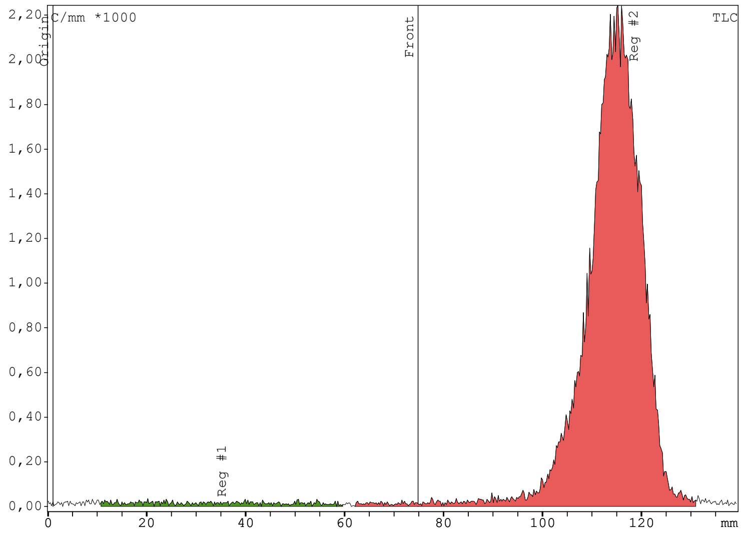

Figure S9: Radio-iTLC chromatograms of [^{99m}Tc]Tc-HYNIC-Chl yield after incubation at 85°C for 15 min (acetonitrile-water (1:1) as a mobile phase). The peak 1 corresponds to [^{99m}Tc]TcO_2 impurity, the peak 2 corresponds to [^{99m}Tc]Tc-HYNIC-Chl, free $^{99m}TcO_4^-$ ions, and other complexes.

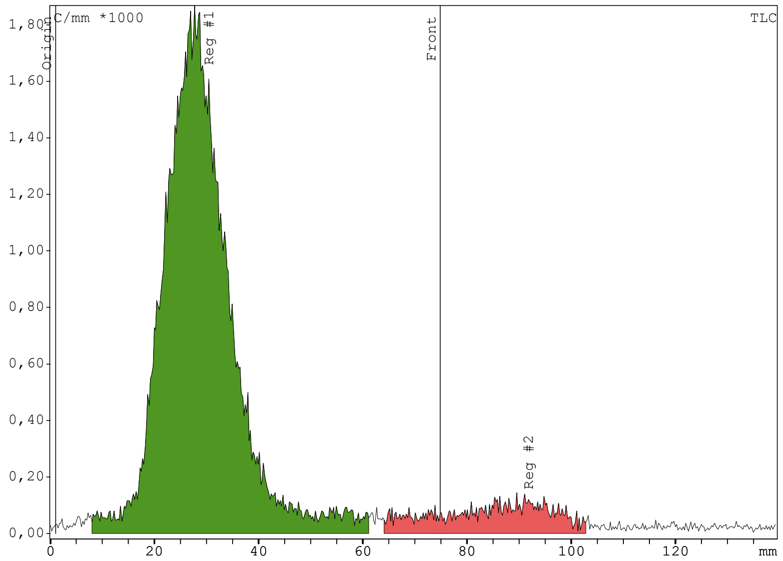

Figure S10: Radio-iTLC chromatograms of [^{99m}Tc]Tc-HYNIC-Chl purity after incubation at 85°C for 15 min and purification using C18 phase (PBS as mobile phase). The peak 1 corresponds to a mixture of [^{99m}Tc]Tc-HYNIC-Chl and [^{99m}Tc]TcO_2 impurity, the peak 2 corresponds to free $^{99m}TcO_4^-$ ions, [^{99m}Tc]Tc-EDDA and [^{99m}Tc]Tc-tricine.

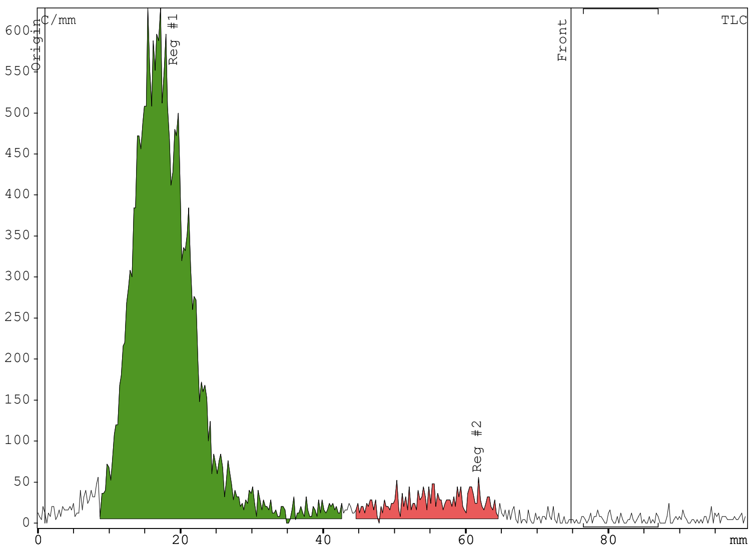

Figure S11: Radio-iTLC chromatograms of [^{99m}Tc]Tc-HYNIC-Chl purity after incubation during 4 h for stability test (PBS as mobile phase). The peak 1 corresponds to a mixture of [^{99m}Tc]Tc-HYNIC-Chl and [^{99m}Tc]TcO_2 impurity, the peak 2 corresponds to free $^{99m}TcO_4^-$ ions, [^{99m}Tc]Tc-EDDA and [^{99m}Tc]Tc-tricine.

Biodistribution data of ^{99m}Tc-HYNIC-Chl

Table S1: The dose-dependent biodistribution of [^{99m}Tc]Tc-HYNIC-Chl (injected doses of 1.2 mg/kg, 6 mg/kg, 12 mg/kg) in Nu/j mice bearing A-431 (epidermoid cancer tumor) xenografts at 2 h post-injection (pi). Data are presented as mean %ID/g ± SD for four animals. Data for the rest of the gastrointestinal tract with contents (GI tract) and the rest of the body are presented as %ID per whole sample.

Organs	^{99m}Tc-HYNIC-Chl 1.2 mg/kg	^{99m}Tc-HYNIC-Chl 6 mg/kg	^{99m}Tc-HYNIC-Chl 12 mg/kg
**Blood**	2.6 ± 0.3	3.5 ± 0.4	3.3 ± 1.0
**Salivary glands**	0.9 ± 0.1[b]	1.1 ± 0.3	1.3 ± 0.1
**Brain**	0.1 ± 0.1	0.13 ± 0.02	0.11 ± 0.01
**Heart**	1.2 ± 0.2	1.6 ± 0.4	1.6 ± 0.2
**Lung**	2.7 ± 0.4	2.9 ± 0.4	2.8 ± 0.4
**Liver**	33.5 ± 5.8	31.6 ± 4.8	26.7 ± 2.5
**Spleen**	7.0 ± 0.8[a]	9.4 ± 0.8	8.1 ± 1.0
**Pancreas**	0.6 ± 0.1	1.0 ± 0.3	0.9 ± 0.3
**Small intestine**	1.6 ± 0.5	2.1 ± 0.8	2.7 ± 0.6
**Large intestine**	1.7 ± 1.1	3.5 ± 2.1	2.3 ± 1.8
**Stomach**	1.3 ± 0.2	1.6 ± 0.3	1.8 ± 0.3
**Kidney**	3.7 ± 0.5	4.5 ± 1.1	3.6 ± 0.3
**Muscle**	0.33 ± 0.04	0.4 ± 0.1	0.5 ± 0.1
**Bone**	2.4 ± 0.1	3.0 ± 0.7	2.8 ± 0.8
**Skin**	1.1 ± 0.2	1.4 ± 0.4	1.5 ± 0.8
**Fat**	0.3 ± 0.1	0.5 ± 0.2	0.6 ± 0.3
**Tumor**	0.7 ± 0.2	0.8 ± 0.2	0.8 ± 0.1
**GI**	8.6 ± 0.6[b]	7.2 ± 1.4	6.6 ± 0.5
**BODY**	10.6 ± 1.6	12.8 ± 2.4	12.2 ± 1.0

a: significant differences between ^{99m}Tc-HYNIC-Chl 1.2 mg/kg and ^{99m}Tc-HYNIC-Chl 6 mg/kg

b: significant differences between ^{99m}Tc-HYNIC-Chl 1.2 mg/kg and ^{99m}Tc-HYNIC-Chl 12 mg/kg

c: significant differences between ^{99m}Tc-HYNIC-Chl 6 mg/kg and ^{99m}Tc-HYNIC-Chl 12 mg/kg

Table S2: Dose-dependent tumor-to-organ ratios of [^{99m}Tc]Tc-HYNIC-Chl (injected doses of 1.2 mg/kg, 6 mg/kg, 12 mg/kg) in Nu/j mice bearing A-431 (epidermoid cancer tumor) xenografts at 2 h post-injection (pi). Data are presented as mean %ID/g ± SD for four animals. Data for the rest of the gastrointestinal tract with contents (GI tract) and the rest of the body are presented as %ID per whole sample.

**Organs**	**^{99m}Tc-HYNIC-Chl 1.2 mg/kg**	**^{99m}Tc-HYNIC-Chl 6 mg/kg**	**^{99m}Tc-HYNIC-Chl 12 mg/kg**
**Blood**	0.3 ± 0.1	0.2 ± 0.1	0.3 ± 0.1
**Salivary glands**	0.8 ± 0.2	0.7 ± 0.1	0.7 ± 0.1
**Brain**	9.3 ± 5.2	5.7 ± 1.5	8.2 ± 1.4
**Heart**	0.6 ± 0.1	0.5 ± 0.1	0.5 ± 0.1
**Lung**	0.3 ± 0.1	0.3 ± 0.1	0.3 ± 0.1
**Liver**	0.02 ± 0.01	0.023± 0.004	0.03 ± 0.01
**Spleen**	0.11 ± 0.03	0.08 ± 0.02	0.11 ± 0.02
**Pancreas**	1.1 ± 0.3	0.8 ± 0.2	1.0 ± 0.3
**Small intestine**	0.5 ± 0.2	0.4 ± 0.1	0.3 ± 0.1
**Large intestine**	0.5 ± 0.4	0.3 ± 0.3	0.5 ± 0.3
**Stomach**	0.5 ± 0.2	0.5 ± 0.2	0.5 ± 0.1
**Kidney**	0.2 ± 0.1	0.16 ± 0.03	0.23 ± 0.03
**Muscle**	2.0 ± 0.4	1.8 ± 0.1	1.8 ± 0.3
**Bone**	0.3 ± 0.1	0.3 ± 0.1	0.3 ± 0.1
**Skin**	0.6 ± 0.2	0.6 ± 0.1	0.6 ± 0.2
**Fat**	2.8 ± 0.7	1.8 ± 0.4	1.9 ± 1.2
